# Synthesis, Characterization and Antitumour Activities of Di-n-Butyl- and Dimethyltin D-(+)-Camphorates

**DOI:** 10.1155/MBD.1998.275

**Published:** 1998

**Authors:** Marcel Gielen, Hassan Dalil, Bernard Mahieu, Dick de Vos, Monique Biesemans, Rudolph Willem

**Affiliations:** 1 Vrije Universiteit Brussel Pleinlaan 2 Brussels B-1050 Belgium; 2 Vrije Universiteit Brussel Department of General and Organic Chemistry Faculty of Applied Sciences Pleinlaan 2 Brussels B-1050 Belgium; 3 Catholic University of Louvain CPMC Louvain-la-Neuve B-1348 Belgium; 4 Medical Department Pharmachemie B. V. Haarlem NL-2003 RN The Netherlands


### SYNTHESIS, CHARACTERIZATION AND ANTITUMOUR ACTIVITIES OF DI-n-BUTYL- AND DIMETHYLTIN D-(+)-CAMPHORATES

Marcel Gielen*[1a], Hassan Dalil[1a], Bernard Mahieu[2], Dick de Vos[3], Monique Biesemans[1], and Rudolph Willem[1]

[1] Vrije Universiteit Brussel, Pleinlaan 2, B-1050 Brussels, Belgium
[a] Department of General and Organic Chemistry, Faculty of Applied Sciences
[b] High Resolution NMR Centre
[2] Catholic University of Louvain, CPMC, B-1348 Louvain-la-Neuve, Belgium
[3] Medical Department, Pharmachemie B. V., NL-2003 RN Haarlem, the Netherlands

**Abstract**
The synthesis and characterization di-n-butyl- and dimethyltin D-(+)-camphorates, respectively compounds **1** and **2**, are reported. Compound **1** displays antitumoural activity *in vitro*, and is more active than cisplatin, 5-fluorouracil and etoposide against seven tumoural cell lines of human origin, but less active than methotrexate and doxorubucin.

Several diorganotin derivatives of dicarboxylic acids are active *in vitro* against human tumoural cell lines[1-6]. Nevertheless, diorganotin derivatives of dicarboxylic acids with proven antitumour activity *in vitro* remain rather rare. The present paper reports the synthesis and characterization of two novel diorganotin dicarboxylates, di-n-butyl- and dimethyltin D-(+)-camphorates, in order to further investigate the influence of the structure of such a dicarboxylate moiety on antitumour properties[7]. The antitumour activity of the di-n-butyltin compound is presented, that of the dimethyltin compound being provided for comparison.
Di-n-butyl- and dimethyltin D-(+)-camphorates, respectively compounds **1** and **2**, were prepared by condensing the appropriate diorganotin oxide with camphoric acid in refluxing toluene/ethanol 4/1 under elimination of the azeotrope water/toluene/ethanol[8-10]. They were recrystallized from ethanol/petroleum ether, m.p. 138-140 °C, yield: 90%, and methylene chloride/hexane, m.p. 258-259 °C, yield: 96%, respectively. The Mössbauer parameters of compounds **1** and **2** are respectively I.S.: 1.39, Q.S.: 3.32, $\Gamma_1$: 1.06, $\Gamma_2$: 0.92 and I.S.: 1.35, Q.S.: 3.89, $\Gamma_1$: 0.93, $\Gamma_2$: 1.02 mm/s. $^1$H and $^{13}$C NMR data are presented in Table 1.

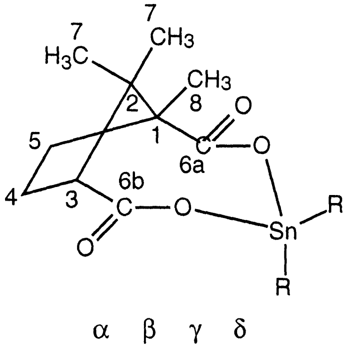

| | | |
|---|---|---|
| | α β γ δ | |
| R = | $CH_2$-$CH_2$-$CH_2$-$CH_3$ | **1** |
| | α | |
| | $CH_3$ | **2** |

The molecular weight of **2** was determined by cryoscopy in camphor (calc. for $C_{12}H_{20}O_4Sn$: 347; found: 343 ± 52 Dalton.

| | $^1H$ | | | $^{13}C$ | |
|---|---|---|---|---|---|
| | **1** | **2** | | **1** | **2** |
| | | | $C_1$ | 56.2 | 56.0 |
| | | | $C_2$ | 46.7 | 46.9 |
| $H_3$ | t: 2.86 [9] | t: 2.85 [9] | $C_3$ | 52.6 | 52.5 |
| $H_{4a}$ | m: 2.22-2.10 | m: 2.22-2.10 | $C_4$ | 23.2 | 22.9 |
| $H_{4b}$ | m: 1.90-1.77 | m: 1.89-1.76 | | | |
| $H_{5a}$ | ddd: 2.53 [12, 8, 8] | ddd: 2.53 [12, 8, 8] | $C_5$ | 32.9 | 32.7 |
| $H_{5b}$ | m: 1.56-1.47 | m: 1.55-1.45 | | | |
| | | | $C_{6a}$ | 186.4 | 186.3 |
| | | | $C_{6b}$ | 184.2 | 184.0 |
| $H_{7a}$ | s: 1.35 | s: 1.33 | $C_{7a}$ | 22.7 | 22.8 |
| $H_{7b}$ | s: 0.87 | s: 0.83 | $C_{7b}$ | 21.5 | 21.5 |
| $H_8$ | s: 1.23 | s: 1.22 | $C_8$ | 22.4 | 21.9 |
| $H_{\alpha\text{-}\beta}$ | m: 1.66-1.51 | s: 0.91 (**81**) | $C_\alpha$ | 25.1 (**594/566**) | 4.4 (**660/630**) |
| | | | $C_\beta$ | 26.8 (**35**) | - |
| $H_\gamma$ | m: 1.48-1.34 | - | $C_\gamma$ | 26.4 (**97**) | - |
| $H_\delta$ | t: 0.87 [7] | - | $C_\delta$ | 13.5 | - |

Table 1: $^1H$ and $^{13}C$ NMR data of compounds **1** and **2** ($CDCl_3$). Chemical shifts in ppm vs. TMS; $^nJ(^1H,^1H)$ coupling constants (between brackets), $^2J(^1H,^{119}Sn)$ and $^nJ(^{13}C,^{119/117}Sn)$ coupling constants in Hz (bold in parentheses); d: doublet; t: triplet; s: singlet; m: complex pattern.

The $^{117}Sn$ NMR chemical shifts $\delta(^{117}Sn)(CDCl_3)$ (ppm) of compounds **1** and **2** are respectively -150.7 and -124.8 ppm. These values are in agreement with the structure proposed, the absence of concentration effect (30 and 100 mg/0.5 ml) excluding any monomer - oligomer equilibrium.

Compounds **1** and **2** were screened *in vitro* against seven tumoural cell lines of human origin: MCF-7 and EVSA-T, two mammary tumours, WiDr, a colon carcinoma, IGROV, an ovarian cancer, M19 MEL, a melanoma, A498, a renal cancer, and H226, a non-small cell lung cancer, as water/ethanol 99/1 solutions.
The results of the antitumoural tests are summarized in table 2 and compared with the inhibition doses $ID_{50}$ obtained for some clinically used reference compounds[11,12], cisplatin, 5-fluorouracil, etoposide, methotrexate and doxorubicin.

| Compounds | MCF-7 | EVSA-T | WiDr | IGROV | M19 MEL | A498 | H226 |
|---|---|---|---|---|---|---|---|
| **1** | 49 | 28 | 100 | 45 | 66 | 49 | 178 |
| **2** | 1342 | 903 | 3504 | 1006 | 1111 | 1548 | 764 |
| Cisplatin | 699 | 422 | 967 | 169 | 558 | 2253 | 3269 |
| 5-Fluorouracil | 750 | 475 | 225 | 297 | 442 | 143 | 340 |
| Etoposide | 2594 | 317 | 150 | 580 | 505 | 1314 | 3934 |
| Methotrexate | 18 | 5 | <3 | 7 | 23 | 37 | 2287 |
| Doxorubicin | 10 | 8 | 11 | 60 | 16 | 90 | 199 |

Table 2. Inhibition doses $ID_{50}$ of compounds **1** and **2** and of some reference compounds[11] against seven tumoural cell lines of human origin

Compound **1** is more active than cisplatin, 5-fluorouracil and etoposide against all cell lines, but less active than methotrexate and doxorubucin. The dimethyltin compound **2** is inactive, as usually[13].

### Acknowledgements
We thank Mrs. I. Verbruggen for recording the NMR spectra. We are grateful to Mr. H. J. Kolker, Dr. J. Verweij, Prof. Dr. G. Stoter, Dr. J. H. M. Schellens, Laboratory of Experimental Chemotherapy and Pharmacology, Department of Medical Oncology, Rotterdam Cancer Institute, NL - 3008 AE, Rotterdam, The Netherlands, for performing the *in vitro* tests. This research was supported by the Belgian "Nationaal Fonds voor Wetenschappelijk Onderzoek" (N.F.W.O. grant nr G.0054.96, M. G.) and the Fund for Scientific Research Flanders (Belgium, grant nr G.0192.98, R. W., M. B.).

**References**

1. R. Willem, M. Biesemans, M. Bouâlam, A. Delmotte, A. El Khloufi, M. Gielen, *Appl. Organomet. Chem.*, **7** (1993), 311.
2. M. Gielen, M. Bouâlam, A. Meriem, B. Mahieu, M. Biesemans, R. Willem, *Heteroatom Chem.*, **3** (1992), 449.
3. M. Gielen, R. Willem, *Anticancer Res.*, **12** (1992), 269.
4. M. Gielen, M. Acheddad, B. Mahieu, R. Willem, *Main Group Met. Chem.*, **14** (1991), 73.
5. A. Meriem, R. Willem, J. Meunier-Piret, M. Gielen, *Main Group Met. Chem.*, **12** (1989), 187.
6. M. Gielen, E. Joosen, T. Mancilla, K. Jurkschat, R. Willem, C. Roobol, J. Bernheim, G. Atassi, F. Huber, E. Hoffmann, H. Preut, B. Mahieu, *Main Group Met. Chem.*, **10** (1987), 147.
7. M. Gielen, *Coord. Chem. Rev.*, **151** (1996), 41.
8. M. Gielen, A. El Khloufi, M. Biesemans, B. Mahieu, R. Willem, *Bull. Soc. Chim. Belg.*, **101** (1992), 243.
9. M. Bouâlam, R. Willem, M. Biesemans, M. Gielen, *Appl. Organomet. Chem.*, **5** (1991), 497.
10. M. Bouâlam, R. Willem, M. Biesemans, B. Mahieu, J. Meunier-Piret, M. Gielen, *Main Group Met. Chem.*, **14** (1991), 41.
11. P. Skehan, R. Storeng, D. Scudiero, A. Monks, J. McMahon, D. Vistica, J. T. Warren, H. Bokesch, S. Kenney, M. R. Boyd, *J. Natl. Cancer Inst.*, **82** (1990), 1107.
12. Y. P. Kepers, G. J. Peters, J. Van Ark-Otte, B. Winograd, H. M. Pinedo, *Eur. J. Cancer,* **27** (1991), 897.
13. M. Gielen, P. Lelieveld, D. de Vos, R. Willem, "Metal Complexes in Cancer Chemotherapy", ed. B. K. Keppler, VCH, Weinheim, 1993, chapter 17, pp. 383 - 390.

**Received: May 28, 1998 - Accepted: July 15, 1998 -**
**Received in revised camera-ready format: July 16, 1998**

